# Whole-brain Functional Networks in Cognitively Normal, Mild Cognitive Impairment, and Alzheimer’s Disease

**DOI:** 10.1371/journal.pone.0053922

**Published:** 2013-01-15

**Authors:** Eun Hyun Seo, Dong Young Lee, Jong-Min Lee, Jun-Sung Park, Bo Kyung Sohn, Dong Soo Lee, Young Min Choe, Jong Inn Woo

**Affiliations:** 1 Department of Neuropsychiatry and Biomedical Research Institute, Seoul National University Hospital, Seoul, Korea; 2 Interdisciplinary Program of Cognitive Science, Seoul National University, Seoul, Korea; 3 Department of Biomedical Engineering, Hanyang University, Seoul, Korea; 4 Department of Nuclear Medicine, Seoul National University Hospital, Seoul, Korea; Beijing Normal University, Beijing, China

## Abstract

The conceptual significance of understanding functional brain alterations and cognitive deficits associated with Alzheimer’s disease (AD) process has been widely established. However, the whole-brain functional networks of AD and its prodromal stage, mild cognitive impairment (MCI), are not well clarified yet. In this study, we compared the characteristics of the whole-brain functional networks among cognitively normal (CN), MCI, and AD individuals by applying graph theoretical analyses to [^18^F] fluorodeoxyglucose positron emission tomography (FDG-PET) data. Ninety-four CN elderly, 183 with MCI, and 216 with AD underwent clinical evaluation and FDG-PET scan. The overall small-world property as seen in the CN whole-brain network was preserved in MCI and AD. In contrast, individual parameters of the network were altered with the following patterns of changes: local clustering of networks was lower in both MCI and AD compared to CN, while path length was not different among the three groups. Then, MCI had a lower level of local clustering than AD. Subgroup analyses for AD also revealed that very mild AD had lower local clustering and shorter path length compared to mild AD. Regarding the local properties of the whole-brain networks, MCI and AD had significantly decreased normalized betweenness centrality in several hubs regionally associated with the default mode network compared to CN. Our results suggest that the functional integration in whole-brain network progressively declines due to the AD process. On the other hand, functional relatedness between neighboring brain regions may not gradually decrease, but be the most severely altered in MCI stage and gradually re-increase in clinical AD stages.

## Introduction

Alzheimer’s disease (AD) is the most common cause of dementia, characterized by progressive cognitive decline including memory impairment. AD process can disrupt neural activities at various levels such as molecular pathways, synapses, neuronal subpopulations, local circuits in specific brain regions, and even higher-order neural networks [1]. Much evidence from neuropathological [2], [3], [4], neuroimaging [5], [6], [7], [8], [9], [10], [11], neurophysiological [12], [13], [14], and neuropsychological studies [15], [16], [17] support the idea of AD as a disconnection syndrome [18], implying that a network-based approach is critical to understand brain alterations and cognitive deficits associated with AD process.

Graph-theoretical analysis, recently applied to brain networks, provides a mathematical and conceptual framework for understanding the brain as a whole network [19]. Graph theory allows capturing various aspects of the brain networks’ global topological organization as well as the local contributions of each area to network function [20]. Many studies report that the human brain has a “small-world” property: much higher local clustering and similar path length compared to matched random networks, providing balance between local specialization and global integration to maximize the efficiency of information processing in brain [21], [22], [23], [24], [25], [26], [27].

Several graph-theoretical analysis studies have been conducted to study the alteration in whole-brain functional network in individuals with AD [27], [28], [29], [30]. Although most of them reported significant AD-related changes in small-world parameters, specific alteration patterns of local clustering and path length reported were controversial. The conflicting results could be attributed to different clinical stages of study subjects. Because brain areas affected by AD pathologies progressively expand as the clinical severity increases [3], network properties might change from the earlier stage to the later stages of AD. Therefore, cautiously selected study samples with distinct disease stages will be helpful to better understand AD-related alterations in functional brain network.

However, the characteristics of the brain network of MCI, a clinical high risk state for AD [31], [32], are not very clear. Very limited numbers of functional brain network studies using magnetoencephalograms (MEG) [33] or resting-state functional magnetic resonance imaging (fMRI) [34] were conducted with MCI individuals reporting inconsistent results. Neuroimaging studies using multivariate statistical analysis or seed-based correlation analysis reported increased medial temporal or prefrontal regions activation with decreased activation in default mode networks (DMN) in MCI patients compared with healthy elderly [35], [36], [37]. They interpreted increased activation as functional compensation. Therefore, it may be possible that the functional brain network of MCI is not necessarily intermediate between those of normal aging and AD. Nevertheless, no study has directly compared the characteristics of whole-brain functional network among cognitively normal (CN) elderly, MCI, and AD.

Mounting evidence suggests that AD begins with a subtle alteration of synaptic function, probably caused by diffusible oligomeric assembles of the amyloid beta protein [38]. Given the increase of synaptic activity leading to an increase of glucose utilization, regional cerebral glucose metabolism (rCMglc), measured by resting [^18^F] fluorodeoxyglucose positron emission tomography (FDG-PET), is a reliable and very sensitive index of synaptic function [39], [40]. To date, however, there have been no graph-theoretical studies using FDG-PET data of the AD or MCI brain.

In this study, we compared the properties of whole-brain functional networks of CN, MCI and AD individuals by applying graph theoretical analyses to FDG-PET data. Additional analyses on AD subgroups were also performed to explore the relationships between whole-brain functional network alterations and clinical severity.

## Materials and Methods

### Subjects

In this study, 94 CN, 183 individuals with MCI, 216 individuals with AD were included. They were recruited among elderly people who participated in a service program for the early detection and management of dementia (two public health centers and one dementia clinic). All subjects lived in the community. A diagnosis of dementia was made according to the criteria of the fourth edition of the Diagnostic and Statistical Manual of Mental Disorders (DSM-IV) [41]. AD was diagnosed according to the probable AD criteria of the National Institute of Neurological and Communication Disorders and Stroke/Alzheimer’s Disease and Related Disorders Association (NINCDS-ADRDA) [42]. Individuals with AD who had an overall clinical dementia rating scale (CDR) [43] of 0.5 or 1.0 were included. MCI was diagnosed according to the current consensus criteria for amnestic MCI [32] and all had an overall CDR of 0.5. All CN subjects received a CDR of 0. The exclusion criteria for all subjects were presence of any serious medical, psychiatric, and neurological disorders that could affect mental function; evidence of focal brain lesions on magnetic resonance image; the presence of severe behavioral or communication problems that would make a clinical examination or FDG-PET scan difficult; left-handedness; an absence of a reliable informant; and, inability to read Korean. Individuals with minor physical abnormalities (e.g., diabetes with no serious complications, essential hypertension, mild hearing loss, or others) were included. The Institutional Review Board of the Seoul National University Hospital, Korea, approved the study, and subjects or their legal representatives gave written informed consent.

### Clinical and Neuropsychological Assessments

All subjects were examined by neuropsychiatrists, who had advanced training in neuropsychiatry and dementia research, according to the Consortium to Establish a Registry for Alzheimer’s Disease (CERAD) clinical, and neuropsychological assessment battery. Standard administration of the CERAD battery was previously described in detail [44], [45], [46]. Reliable informants were interviewed to acquire the accurate information regarding the cognitive, emotional and functional changes as well as the subject’s medical history. A panel consisting of four psychiatrists with expertise in dementia research made the clinical decisions and diagnosed dementia, and this panel also reviewed all available raw data resulting from clinical evaluations.

### PET Image Acquisition and Preprocessing

PET studies were performed using the ECAT EXACT 47 scanner (Siemens-CTI, Knoxville, Tenn., USA), which has an intrinsic resolution of 5.2-mm full width at half maximum (FWHM) and the images of 47 contiguous transverse planes with a 3.4-mm thickness for a longitudinal field of view of 16.2 cm. Mean intervals between PET scan and clinical assessment were 10 days. All of the [^18^F] FDG PET scans were performed in a dimly lit room with minimal auditory stimulation during both the injection and PET scanning. Subjects took a supine position with their eyes closed during the scanning to minimize the confounding effects of any activity. More specific information for image acquisition procedures were previously described in detail [47], [48].

Imaging data were preprocessed using Statistical Parametric Mapping 2 (SPM2) (Institute of Neurology, University College of London, UK) implemented in the Matlab (Mathworks Inc, USA). Before statistical analysis, all images were spatially normalized to the Montreal Neurological Institute (MNI, McGill University, Montreal, Canada) space. Normalized images were smoothed by convolution using an isotropic Gaussian kernel with 12 mm full width at half maximum.

### Construction of Brain Metabolic Networks Using Graph Theoretical Approach

**Figure 1:**
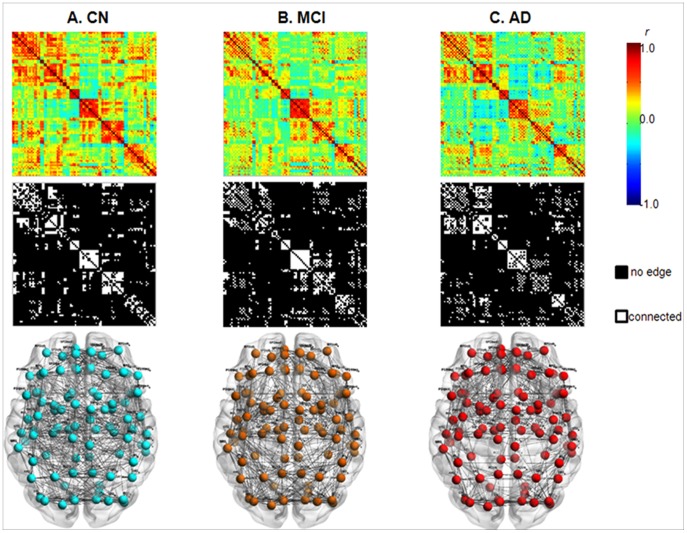
Whole-brain functional networks in CN, MCI, and AD. The first row displays the correlation matrices obtained from partial correlation coefficients (indicated by color bar, range from −1.0 to 1.0) between 90 regions of interests controlling for age, education and gender. The second row displays binary matrices thresholded at a fixed density 15%. The third row illustrates the corresponding brain connectivity graph from the binary matrices. Brain connectivity graphs were visualized using the BrainNet viewer (NKLCNL, Beijing Normal University). Abbreviations for the regions are expanded in Table 1. CN = cognitively normal; MCI = mild cognitive impairment; AD = Alzheimer’s disease.

In graph theory, a network is a set of nodes and edges between pairs of nodes [20]. In our study, nodes were represented by 90 regions of interest (ROI) defined using automated anatomical labeling (AAL) template [49], which has been broadly used in brain network studies [21], [25], [28], [30], [50]. In AAL template, 45 ROIs in each hemisphere except cerebellum were defined based on an anatomical parcellation of MNI-normalized single-subject high-resolution T1 volume [49]. The whole-brain functional networks were constructed with those 90 cortical and subcortical mean values of rCMglc (Table 1). The rCMglc of each ROI were globally normalized with respect to mean metabolic rate for glucose in each individual’s whole brain. We selected global normalization procedure because it has higher signal-to-noise ratio compared to the cerebellar count normalization method [51] and correlation coefficients are obtained separately for each diagnostic group. Interregional correlation matrix (90X90) was acquired by partial correlation analysis controlling for age-, gender-, and education- effects. These partial correlation coefficients between every pair of ROIs represent edges, in other words, functional connections between nodes. To avoid complicated statistical descriptions in the following network analysis, our graph theoretical analysis is confined to a simple undirected and unweighted binary matrix (Figure 1). The interregional correlation matrix was then transformed into a binary matrix using a fixed density threshold method. Density is the fraction of present connections to possible connections [20]. Fixed density threshold method ensures the graphs from three diagnostic groups have the same number of edges [52]. As there is no gold standard for a single threshold, we applied a wide range of density (D), i.e., 6%≤ *D* ≤40% with an incremental interval of 1% and repeated the full analysis for each density. This range of density was selected to estimate small-world properly as suggested in previous studies [52], [53].

**Table 1:** Anatomical parcellation defined by automated anatomical labeling atlas and abbreviations for the regions.

Abbreviations	Regions
PreCG	Precental gyrus
SFGdor	Superior frontal gyrus, dorsolateral
SFGorb	Superior frontal gyrus, orbital part
MFG	Middle frontal gyrus
MFGorb	Middle frontal gyrus, orbital part
IFGoperc	Inferior frontal gyrus, opercular part
IFGtriang	Inferior frontal gyrus, triangular part
IFGorb	Inferior frontal gyrus, orbital part
ROL	Rolandic operculum
SMA	Supplementary motor area
OLF	Olfactory cortex
SFGmed	Superior frontal gyrus, medial
SFGmedO	Superior frontal gyrus, medial orbital
REC	Gyrus rectus
INS	Insula
ACC	Anterior cingulate and paracingulate gyri
MCC	Median cingulate and paracingulate gyri
PCC	Posterior cingulate gyrus
HIP	Hippocampus
PHG	Parahippocampal gyrus
AMYG	Amygdala
CAL	Calcarine fissure and surrounding cortex
CUN	Cuneus
LING	Lingual gyrus
SOG	Superior occipital gyrus
MOG	Middle occipital gyrus
IOG	Inferior occipital gyrus
FFG	Fusiform gyrus
PoCG	Postcentral gyrus
SPG	Superior parietal gyrus
IPL	Inferior parietal, but supramarginal andangular gyri
SMG	Supramarginal gyrus
ANG	Angular gyrus
PCUN	Precuneus
PCL	Paracentral lobule
CAU	Caudate nucleus
PUT	Lenticular nucleus, putamen
PAL	Lenticular nucleus, pallidum
THA	Thalamus
HES	Heschl gyrus
STG	Superior temporal gyrus
STGP	Temporal pole: superior temporal gyrus
MTG	Middle temporal gyrus
MTGP	Temporal pole: middle temporal gyrus
ITG	Inferior temporal gyrus

### Network Analysis

Network parameters used in the current study are listed with mathematical definitions in Table S1. Two fundamental network parameters are the clustering coefficient and the characteristic path length. Clustering coefficient *C_i_* of node *i* indicates the likelihood of the neighboring nodes to be connected to each other. Clustering coefficient for a network (*C_p_*) is the average *C_i_* from entire nodes in the network, which quantifies the extent of local interconnectivity of information transfer in a network [20], [54]. Characteristic path length (*L_p_*) is the mean minimum number of edges of the shortest path connecting any two nodes in a network, which quantifies the extent of functional integration of a network [20], [54]. The distinctive combination of high *C_p_* with short *L_p_* is the key property of small-world network [54]. Matched random networks with the same node degree distribution as the brain networks in the present study were generated 1000 times repeatedly and mean value of clustering coefficients (*C_p_^rand^*) and characteristic path length *(L_p_^rand^*) were used as representative parameters of random network. A network is considered as a small-world network if it shows much higher *C_p_* (γ = *C_p_^real^*/*C_p_^rand^* ≫1) while similar *L_p_* (λ = L*_p_^real^*/*L_p_^rand^*_≈_1) in comparison with the matched random network [54]. That is, small-world index *σ* = γ/λ is greater than 1. Small-worldness tests were done repeatedly over a range of density (i.e., 6%≤ *D* ≤40%).

For the local nodal characteristics, we employed betweenness centrality. Betweenness centrality, *B_i_* of a node *i* is defined as the number of shortest paths between any two nodes that run through node i, which quantifies how much information might traverse the node, presuming that optimal paths are used [20], [55]. *B_i_* of a node i was used to determine candidate hubs in a network. The *B_i_* was normalized as *b_i_* = *B_i_*/averaged *B_i._* for all nodes of the entire network. The nodes that have high *b_i_* (>1.5) are considered as functional hubs of a network. The *b_i_* of each node was calculated at a fixed density 15%. Certain density threshold, which ensures all of 90 ROIs are included and false-positive paths are minimized, was determined as a fixed density. The lowest density where the largest component size was 90 (*i.e.,* all connected nodes included) was density 15% in the present study.

Calculations of these network parameters were performed using ‘Brain connectivity analysis software’ (http://www.brain-connectivity-toolbox.net) [20] and the MatlabBGL package (http://www.stanford.edu/~dgleich/program/matlab_bgl/). Hubs were visualized with the BrainNet Viewer (http://www. nitrc.org/projects/bnv/).

### Statistical Analysis

#### Differences in network parameters

Between-group differences in network parameters (*C_p,_ L_p,_* σ and *b_i_*) were tested using a nonparametric permutation test with 5000 repetitions [52], [56]. To test if the observed group difference occurred by chance (the null hypothesis), we randomly reassigned each participant’s 90 ROI rCMglc values to one of two groups. After the randomization procedure, the interregional correlation matrix was calculated again and a set of binary matrices was also obtained over the same density threshold range as in the real brain networks. *C_p,_ L_p,_* σ and *b_i_* were calculated in each network separately, and then between-group differences in the network parameters were obtained. This procedure was repeated 5000 times and the 95 percentile scores of each difference-distribution were considered as the critical values (*p*<0.05, one-tailed). This nonparametric permutation test procedure was performed repeatedly at 6%≤ D ≤40% (35 times). We did not make any adjustment for multiple comparisons because we tried to explore the general trends of between-group differences through the wide range of density level rather than to put a separate and specific interpretation on the result at a certain density level.

#### Severity subgroup analysis for AD

AD group was divided into two severity subgroups according to CDR score: ‘very mild AD (CDR = 0.5)’ and ‘mild AD (CDR = 1.0)’. Network construction for each subgroup and between-subgroup comparisons were performed using the procedures described above.

## Results

### Subject Characteristics

Demographic and clinical characteristics of participants are summarized in Table 2. There were no significant differences between diagnostic groups in age, gender, and education. In terms of neuropsychological performance, all of the test scores were significantly different between any two groups. For the subgroups of AD, 121 were very mild AD (CDR = 0.5) and 95 were mild AD (CDR = 1.0).

**Table 2:** Demographic and clinical characteristics.

	CN	MCI	AD
	(*n* = 94)	(*n* = 183)	(*n* = 216)
Age (SD), yrs	70.1 (6.2)	71.1 (6.6)	70.8 (8.0)
Education (SD), yrs	9.3 (4.9)	8.8 (4.9)	7.9 (5.6)
Gender (*n* of male/female)	32/62	54/129	63/153
CDR (*n* of 0/0.5/1.0)	94/0/0	0/183/0	0/121/95
CDR SOB	0 (0)	1.25 (0.62)^a^	4.25 (1.69)a, b
MMSE score (SD)	26.5 (2.4)	22.6 (4.0)^a^	17.4 (5.0)a, b
Neuropsychological Test			
Animal fluency	16.0 (4.7)	11.4 (3.7)^a^	8.4 (3.8)a, b
Boston naming	11.7 (2.2)	9.4 (2.8)^a^	7.5 (3.4)a, b
Word list learning	18.9 (4.4)	12.6 (4.4)^a^	8.5 (4.3)a, b
Word list recall	6.8 (1.9)	3.1 (2.0)^a^	1.1 (1.4)a, b
Word list recognition	9.3 (1.0)	6.8 (2.3)^a^	4.5 (2.8)a, b
Constructional praxis	10.3 (1.1)	9.5 (1.8)^a^	8.3 (2.3)a, b
Constructional recall	7.0 (2.8)	3.2 (2.9)^a^	1.5(2.0)a, b

Values are given as mean (standard deviation) except gender and CDR.

a Significant compared to CN (*p*<0.05);

a, b Significant compared to MCI (*p*<0.05).

CN = cognitively normal; MCI = mild cognitive impairment; AD = Alzheimer’s disease; CDR = Clinical dementia rating; CDR SOB = sum of boxes of the clinical dementia rating; MMSE = mini mental status examination.

### Small-world Properties and their Alterations in MCI and AD

**Figure 2:**
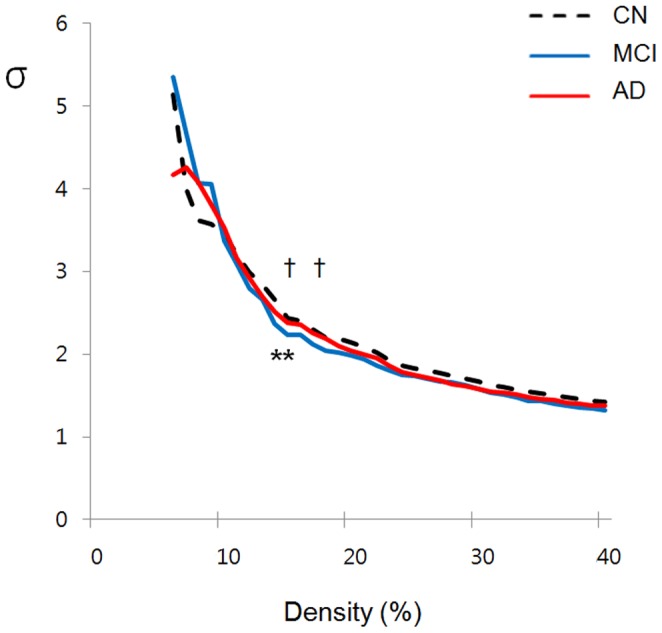
Small-world index of functional brain networks in CN, MCI, and AD. σ = Small-world index; CN = cognitively normal; MCI = mild cognitive impairment; AD = Alzheimer’s disease. *p<0.05 for CN vs. MCI (significant at density of 14%∼15%); ^†^ *p*<0.05 for AD vs. MCI (significant at 15% and 18%).

**Figure 3:**
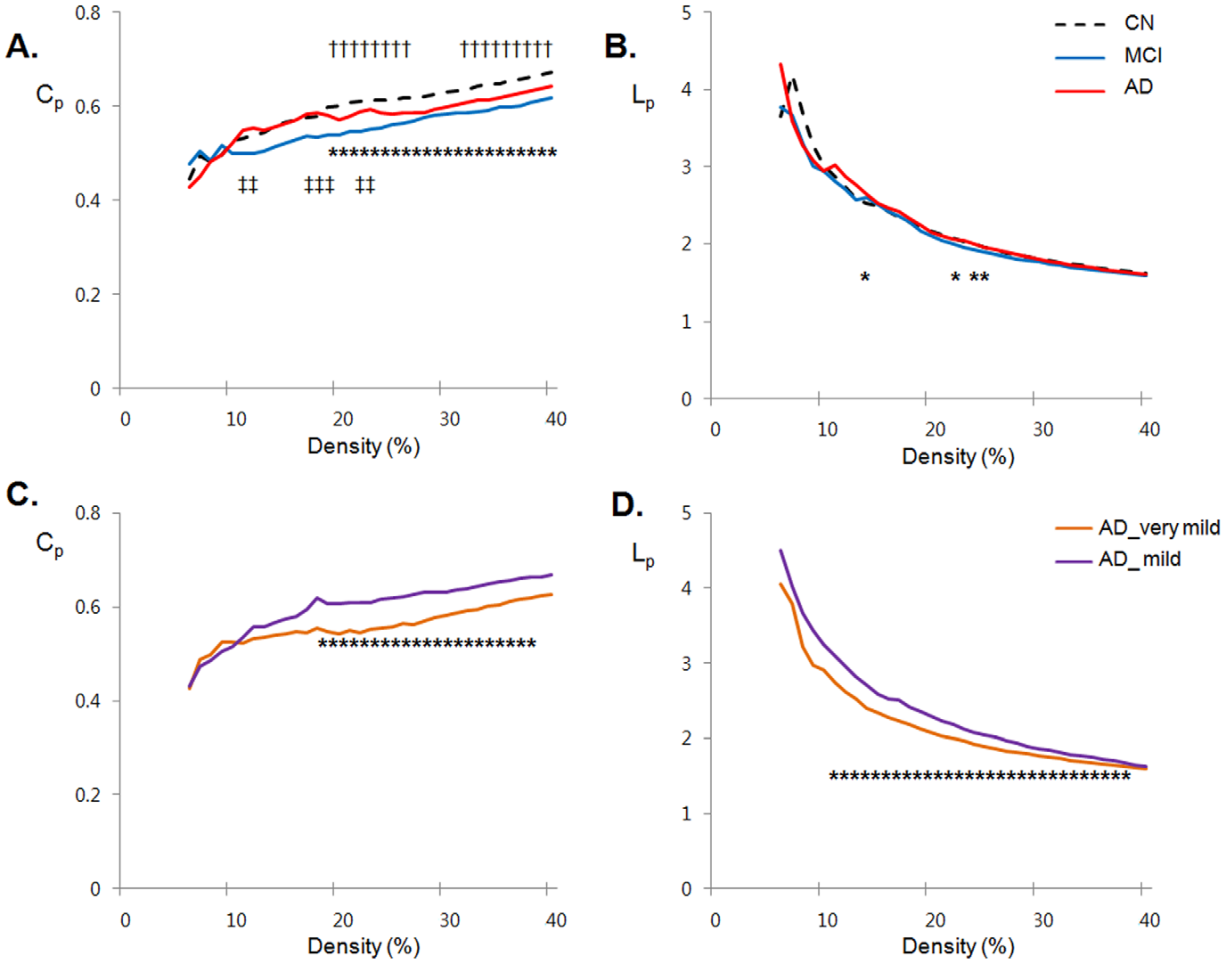
AD- and MCI- related alterations in small-world parameters. A. The clustering coefficients (*C_p_*) from cognitively normal (CN, black dotted line), mild cognitive impairment (MCI, blue line) and Alzheimer’s disease (AD, red line). *p<0.05 for CN vs. MCI (significant at density of 19%∼40%).; ^†^ *p*<0.05 for CN vs. AD (significant at 19%∼26%, and 32%∼40%); ^‡^p<0.05 for MCI vs. AD (significant at 11%∼12%, 17%∼19% and 22%∼23%). **B.** The *L_p_* from CN, MCI and AD. *p<0.05 for MCI vs. AD (significant at 13%, 23%, and 25%∼26%). **C.** The *C_p_* from AD with clinical dementia rating (CDR) 0.5 (AD_very mild, orange line), and AD with CDR 1.0 (AD_mild, purple line). *p<0.05 for AD_very mild vs. AD_mild (significant at 18%∼38%). **D.** The *L_p_* from AD_very mild and AD_mild. *p<0.05 for AD_very mild vs. AD_mild (significant at 11%∼39%).

All three groups demonstrated small-world property (σ>1) over an entire range of density (Figure 2). Between-group comparisons revealed that σ was not significantly different among groups over a wide range of density except 14%–15% (greater in CN compared to MCI) or 15% and 18% (greater in AD compared to MCI). On the other hand, in terms of individual parameters, both AD and MCI group had lower *C_p_* than CN over a wide range of density (Figure 3A). MCI showed significantly lower *C_p_* than AD at certain densities (11%∼12%, 17%∼19% and 22%∼23%). In terms of *L_p_*, CN, MCI, and AD did not show significant between-group differences, but AD had significantly longer *L_p_* than MCI at certain densities (13%, 23%, 25%, and 26%) (Figure 3B).

In subgroup analysis, mild AD subgroup had increased *C_p_* and longer *L_p_* than very mild AD subgroup (Figure 3C and 3D), while there were no group differences in *σ* over entire density.

### Functional Hubs and their Connectivity Alterations in MCI and AD

**Figure 4:**
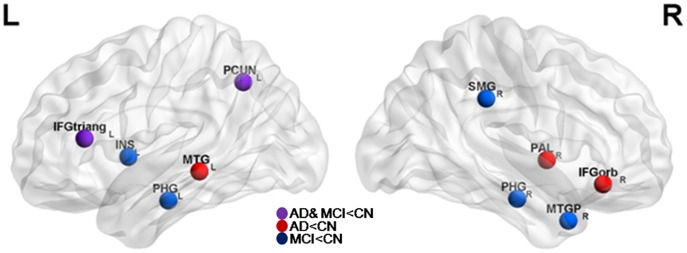
AD- and MCI -related alterations in hub regions. Hubs were visualized using the BrainNet viewer (NKLCNL, Beijing Normal University). Purple circle indicates the regions where both Alzheimer’s disease (AD) and mild cognitive impairment (MCI) networks showed decreased betweenness centrality compared with cognitively normal (CN). Red circle indicates the regions where AD network additionally showed decreased betweenness centrality compared with CN. Blue circle indicates the regions where MCI network additionally showed decreased betweenness centraility compared with CN. Abbreviations for the regions are expanded in Table 1. L = left; R = right.

Several brain regions were identified as functional hubs in CN. They were mainly located in unimodal and multimodal association cortex and paralimbic regions, such as the prefrontal cortex, posterior parietal cortex, lateral temporal cortex and parahippocampal region (Figure S1 and Table S2). Further between-group comparisons revealed that both MCI and AD groups had significantly lower *b_i_* in the left inferior frontal triangular part (IFGtrang_L) and the left precuneus (PCUN_L) compared to CN. The AD group additionally had significantly lower *b_i_* in the bilateral inferior frontal gyrus and the left middle temporal gyrus. MCI group additionally showed significant *b_i_* decrease in the parahippocampal gyrus (Figure 4 and Table S2).

**Figure 5:**
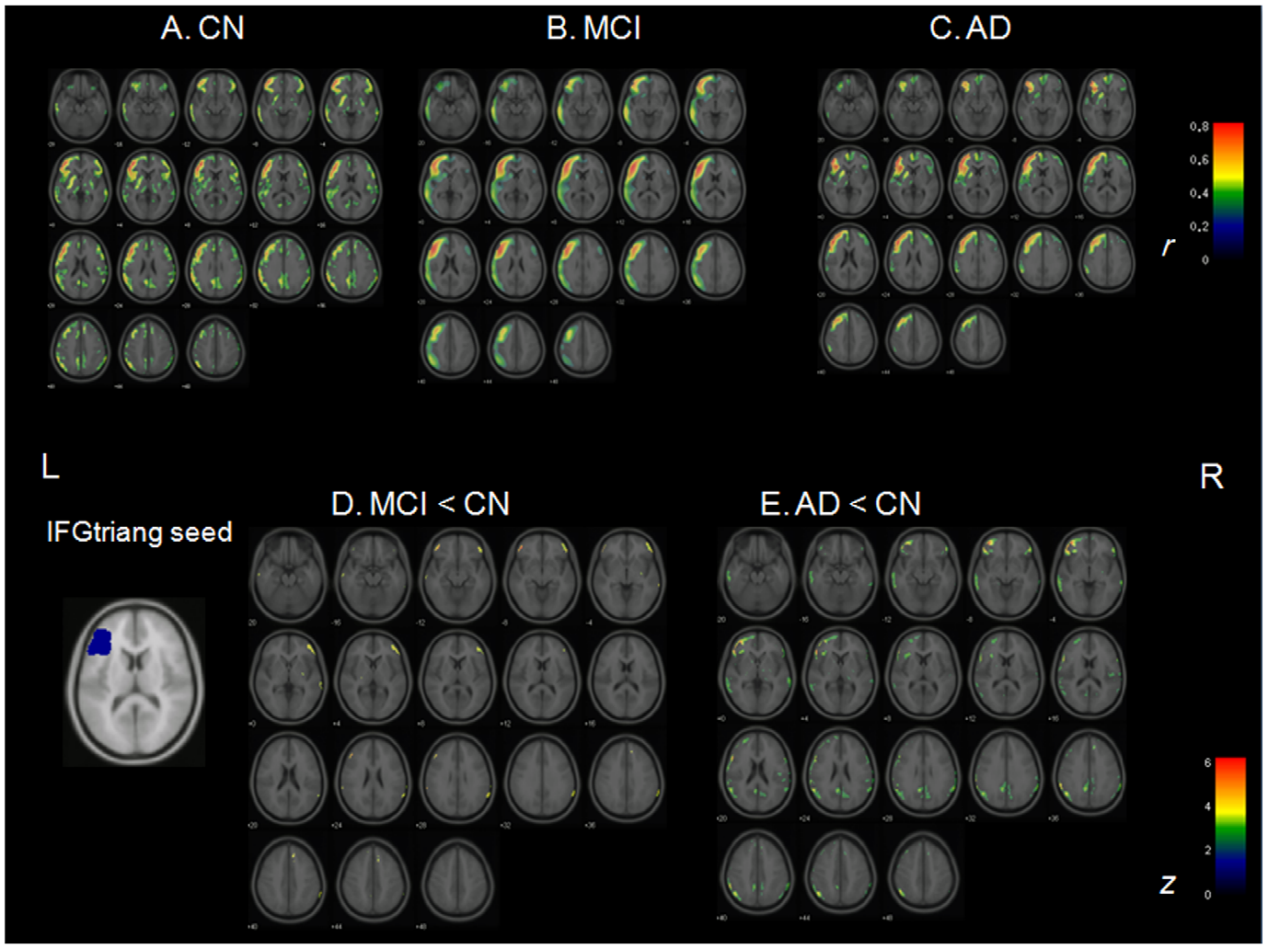
Connectivity associated with left IFGtriang seed and connectivity reductions in CN, MCI, and AD. A. Correlation coefficients map (R-map) showing connectivity associated with left IFGtriang seed in CN. **B.** R-map showing connectivity associated with left IFGtriang seed in MCI. **C.** R-map showing connectivity associated with left IFGtriang seed in AD. **D.** Z-statistics map showing reduced connectivity with left IFGtriang seed in MCI compared to CN. **E.** Z-statistics map showing reduced connectivity left IFGtriang seed in AD compared to CN (*p*<0.05, FDR-corrected). IFGtriang = triangular part of inferior frontal gyrus; CN = cognitively normal; MCI = mild cognitive impairment; AD = Alzheimer’s disease; L = left; R = right.

**Figure 6:**
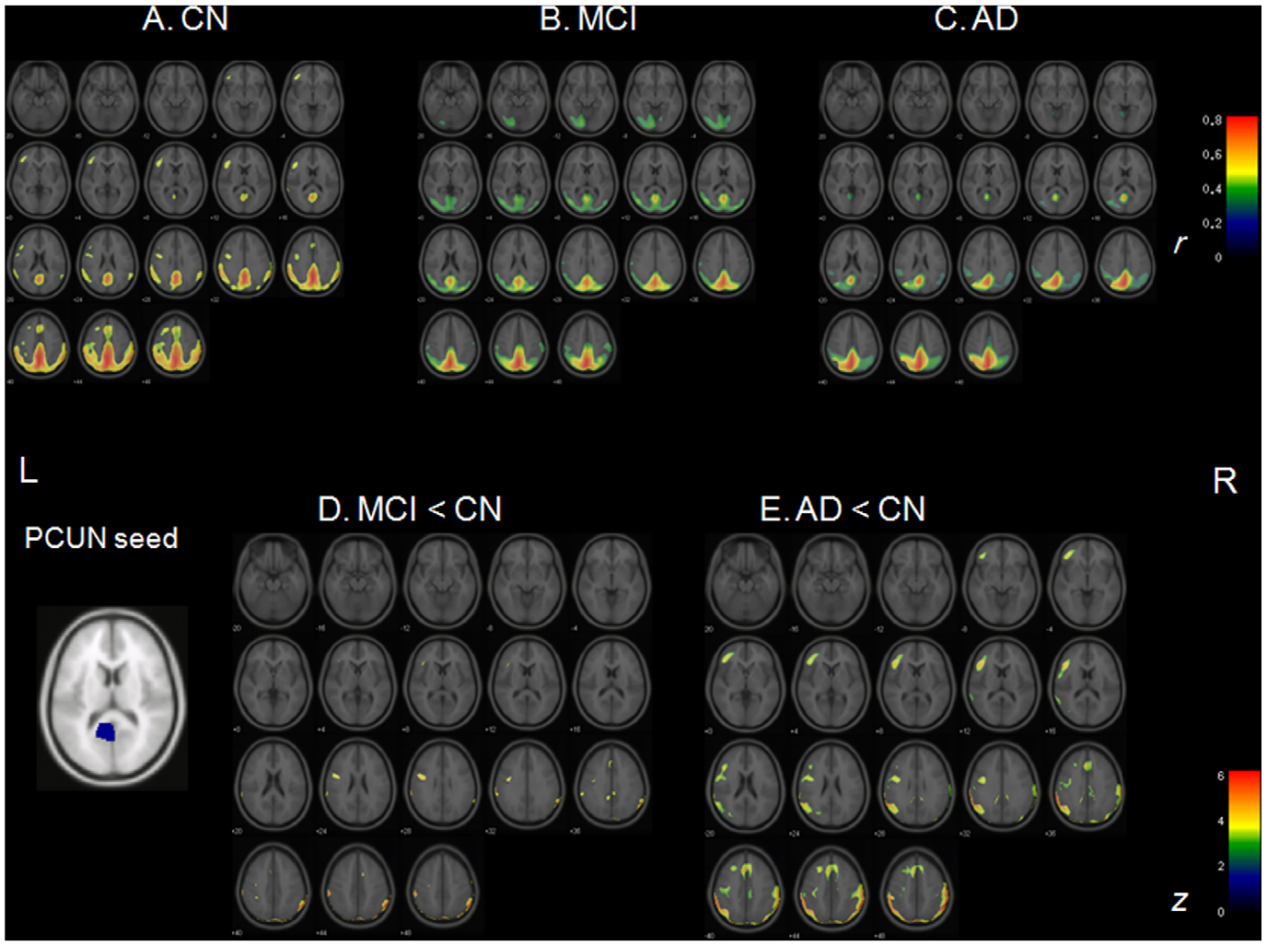
Connectivity associated with left PCUN seed and connectivity reductions in CN, MCI, and AD. A. Correlation coefficients map (R-map) showing connectivity associated with left PCUN seed in CN. **B.** R-map showing connectivity associated with left PCUN seed in MCI. **C.** R-map showing connectivity associated with left PCUN seed in AD. **D.** Z-statistics map showing reduced connectivity with left PCUN seed in MCI compared to CN. **E.** Z-statistics map showing reduced connectivity left PCUN seed in AD compared to CN (*p*<0.05, FDR-corrected). PCUN = precuneus; CN = cognitively normal; MCI = mild cognitive impairment; AD = Alzheimer’s disease; L = left; R = right.

To investigate more detailed connectivity alteration patterns associated with the identified hubs, seed ROI-based interregional correlation analysis was performed. We selected the IFGtrang_L and PCUN_L as seed ROIs because their *b_i_*s significantly decreased in both MCI and AD groups compared with CN. Extracted rCMglc values of those two ROIs were used as covariates to find regions showing significant voxel-wise positive correlations across three groups using Pearson’s correlation coefficient (*p*<0.05, corrected). Figure 5A∼5C shows interregional connectivity with IFGtrang_L seed. These correlation coefficients maps (R-map) were compared between groups using Fisher’s r-to-z transformation, which ensures approximate normal distribution, using *Z_i_* = 1/2 log [(1+*r_i_*)/(1–*r_i_*)]. These z-values were compared between groups by Z statistics using 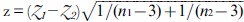. R-map comparisons showed reduced connectivity of IFGtrang_L mainly with the right lateral prefrontal regions in MCI, and mainly with the bilateral lateral prefrontal regions and parietal regions in AD, compared with CN (Figure 5D and 5E). Figure 6A∼6C shows interregional connectivity with PCUN_L seed in all subjects (*p*<0.05, corrected). R-map comparisons showed reduced connectivity of PCUN_L with the left inferior frontal gyrus, bilateral middle cingulate cortex, and right parietal regions in MCI, and with more extensive areas in AD group (Figure 6D and 6E).

## Discussion

This is the first graph theoretical study using FDG-PET data to investigate the characteristics of whole-brain functional network in CN, MCI, and AD. To date no study has directly compared the characteristics of whole-brain functional network among these three groups. Our study revealed three main findings: (1) Whole-brain functional networks of CN, MCI, and AD all showed small-world property; (2) local clustering (*C_p_*,) of network was lower in both MCI and AD compared with CN, while small-world index (σ) itself and path length (*L_p_*) were not different among the three groups. Then, MCI showed lower level of local clustering than AD; (3) subgroup analyses for AD showed that very mild AD had lower local clustering and shorter path length compared to mild AD.

### Altered Global Properties of MCI and AD Functional Brain Networks

Although whole-brain functional networks of MCI and AD showed small-world properties like that of CN, local clustering reflecting the degree of closeness between neighboring brain regions was lower in MCI and AD compared to CN network. Interestingly, MCI showed a more severe alteration in local clustering than AD. This finding was rather unexpected because MCI has been regarded as an intermediate state between CN and AD dementia. The novel finding may be explained by the patterns of regional brain hypometabolism in MCI and AD. MCI individuals show localized hypometabolism pattern typically involving the posterior cingulate/precuneus [57], [58], [59] or medial temporal areas [60], [61], with relatively intact other neighbor regions. In contrast, AD patients have more diffused hypometabolism involving the lateral temporo-parietal and frontal cortices [59], [62] as well as the regions impaired in MCI stage. As the local clustering (*C_p_*,) of network is based on correlation between adjacent brain regions, MCI with scattered and focal impairment pattern shows a more decreased level of local clustering than AD with more diffuse impairment. This implies that hypometabolism extends to wider brain regions as AD progresses, which subsequently results in higher interregional correlation and restoration of clustering coefficient in more advanced stage. To investigate whether such trend is observed even within the AD group, we conducted additional analyses dividing overall AD into very mild (less severe) and mild (more severe) subgroups according to CDR rating. The subgroup analyses revealed that very mild AD had more decreased local clustering than mild AD, supporting the idea that the progression pattern of local clustering impairment from MCI to AD also exists within AD group. Overall, this early disruption and later restoration of clustering coefficient in MCI and early AD implies that this parameter should be used only for the understanding functional relationship between adjacent brain regions, but not for understanding the degree of functional or cognitive impairment by AD pathological process.

In terms of path length, there is no apparent difference of path length (*L_p_*) between CN, MCI and AD at most density levels. AD group, however, showed slightly longer trends for path length, compared to MCI, even with significant differences at a few densities (13%, 23%, 25%, and 26%). Through subgroup analyses for AD, we also found that mild AD patients had significantly longer path length than very mild patients. Taking these findings together, path length seems to gradually increase with the advance of AD pathological process. This is also in accordance with previous reports which indicated that long-distant inter-regional functional connections were relatively spared in MCI, before impairment in AD [33], [37], [63]. In addition, increased path length may be associated with the alteration of long cortico-cortical white matter tracts by AD process [6], [64], because structural connectivity could place physical constraints on functional interactions between nodes [65]. When a long axonal tract directly connecting two distant regions is impaired by AD pathology, information has to be transferred only in a roundabout way through several steps of synapses resulting in a longer path length between the nodes.

As mentioned in the introduction, specific alteration patterns of local clustering and path length reported by earlier functional brain network studies for AD were controversial, and it is hard to find a certain solid trend from the findings: local clustering in the AD network was either unchanged [27], [28] or reduced [29], [30]. Path length between nodes in the AD network was either unchanged [30], or altered into a longer path length [27], [29] or shorter path length [28]. These conflicting results may be attributed to different AD clinical stages of study subjects, given the progressive nature of AD involvement. However, direct comparison between previous reports and our results are difficult because most previous studies did not clearly define the clinical stages of the AD group. The mini mental status examination (MMSE) score of subjects included in previous studies ranged widely (e.g., 12 to 29). They appear to include heterogeneous clinical stages, i.e., very mild to moderate. One recent small world brain network study focused on homogeneous moderate AD patients and reported clear increases in both local clustering and path length [66], very similar to our findings in relatively more advanced AD (mild AD). Studies using structural MRI or diffusion tensor imaging also consistently demonstrated that AD brain network had longer path length and higher clustering with neighbors, compared to the network of CN [50], [52], [67].

### Altered Local Properties of MCI and AD Whole-brain Functional Networks

MCI and AD showed significantly decreased normalized betweenness centrality in several hubs of association cortex compared to CN. The IFGtrang_L and PCUN_L regions, in particular, showed decreased centrality in both MCI and AD. Additional seed ROI-based analyses demonstrated that areas with which IFGtrang_L and PCUN_L ROI had reduced connectivity were more extensive in AD than in MCI (Figure 5 and 6), implying a progressive pattern of network alteration during the AD process. Beside the two regions, there were several additional regions which showed decreased centrality in the MCI or AD network. MCI network showed an additional centrality decrease in the bilateral parahippocampal gyrus, possibly related to episodic memory impairments. AD network showed an additional centrality decrease in the frontal and the temporal regions, possibly related to the impairments of multiple cognitive domains of AD dementia state. These findings indicate that the precuneus, inferior frontal gyrus, insular, parahippocampal gyrus, and middle temporal gyrus are less effective as hubs in MCI and AD than in CN network. These regions generally correspond to the parts of the DMN known to show reduced functional connectivity in MCI or AD [9], [10], [68], [69]. Moreover, the DMN or cortical hubs observed in young adults were anatomically similar to the cortical areas with amyloid deposition in AD patients [70], [71]. Functional impairment of hubs within DMN in MCI and AD patients suggests that cognitive impairments in these individuals may be related to the failure of efficient information transfer among the brain regions given that the hubs serve to integrate diverse informational sources and local networks [70], [72].

### Methodological Considerations

It should be noted that we used a fixed density method rather than the correlation coefficient threshold. As He and colleagues noted, applying the same threshold of correlation coefficients to the matrices of individual groups would result in networks with different edge numbers [52]. A resultant network difference would not simply reflect the true discrepancy of the topological network structures between study groups. Using fixed density threshold ensures the graphs from three diagnostic groups to have the same number of edges. However, fixed density threshold strategy may also influence the results, as it would allow the ROI pairs which have weaker correlation (e.g., AD network) to have an edge in the binary matrix. To account for this issue, we calculated the same network parameters repeatedly with a range of correlation coefficient thresholds (0.10≤ *r* ≤0.70 with increments of 0.05). Group-comparison results were largely similar to those based on fixed density threshold strategy. This supports the validity of the fixed density method in comparing pure global topology of network without losing much of the biological meaning.

### Limitations and Future Questions

There are several limitations and future directions to be addressed. First, a cross-sectional design was used in the current study. Based on the present findings, the diagnostic or prognostic value of network parameters needs to be further explored longitudinally. Second, our findings from the functional brain network study showed close overlap with those from some previous structural network studies. The integration of multimodal imaging study could provide the relationship between functional and anatomical brain networks (e.g., how the functional brain network changes are associated with underlying anatomical change in MCI and AD). Third, we constructed a network using 90 nodes. Although the small-world property was robust and results remained largely similar regardless of the parcellation method [28], [73], network definition by different parcellation (e.g., higher-resolution) might lead to different implications. Finally, unweighted and binary matrix was used in the study. To capture important information from functional brain network as a complex system, more advanced network analysis approach will be needed in the future.

### Conclusion

We compared the characteristics of whole-brain functional network among CN, MCI, and AD through graph theoretical analysis for brain glucose metabolism. Our results for global functional network properties indicated that although the overall small-world property is preserved in MCI and AD, individual parameters of the network are altered with unique patterns of changes: The functional integration reflected in path length appears to progressively decrease by AD process. On the other hand, functional relatedness between neighboring brain regions reflected in local clustering coefficient seems not gradually impaired, but the most severely altered in MCI stage and gradually re-increased in clinical AD stages. In addition, the results on functional impairment of hubs regionally associated with DMN in MCI and AD suggested that cognitive impairments of these individuals may be related the failure of efficient information transfer among brain regions given that the hubs serve to integrate diverse brain informational sources.

## Supporting Information

Figure S1 **Functional hubs in CN, MCI, and AD.** Normalized betweenness centrality >1.5 were considered as a hub. Node size corresponds with its value of normalized betweenness centrality. Hubs were visualized using the BrainNet viewer (NKLCNL, Beijing Normal University). CN = normal control; MCI = mild cognitive impairment; AD = Alzheimer’s disease.
(TIF)

Table S1 **Mathematical definitions of network parameters used in the study** [20].
(DOC)

Table S2 **Functional hubs and nodal characteristics in normal control, mild cognitive impairment, and Alzheimer’s disease.**
(DOC)
