# Supplementary figures and images for: Whole-brain Functional Networks in Cognitively Normal, Mild Cognitive Impairment, and Alzheimer’s Disease
Source: PLoS One. 2013 Jan 15;8(1):e53922. doi: 10.1371/journal.pone.0053922 (PMC3545923; doi:10.1371/journal.pone.0053922)

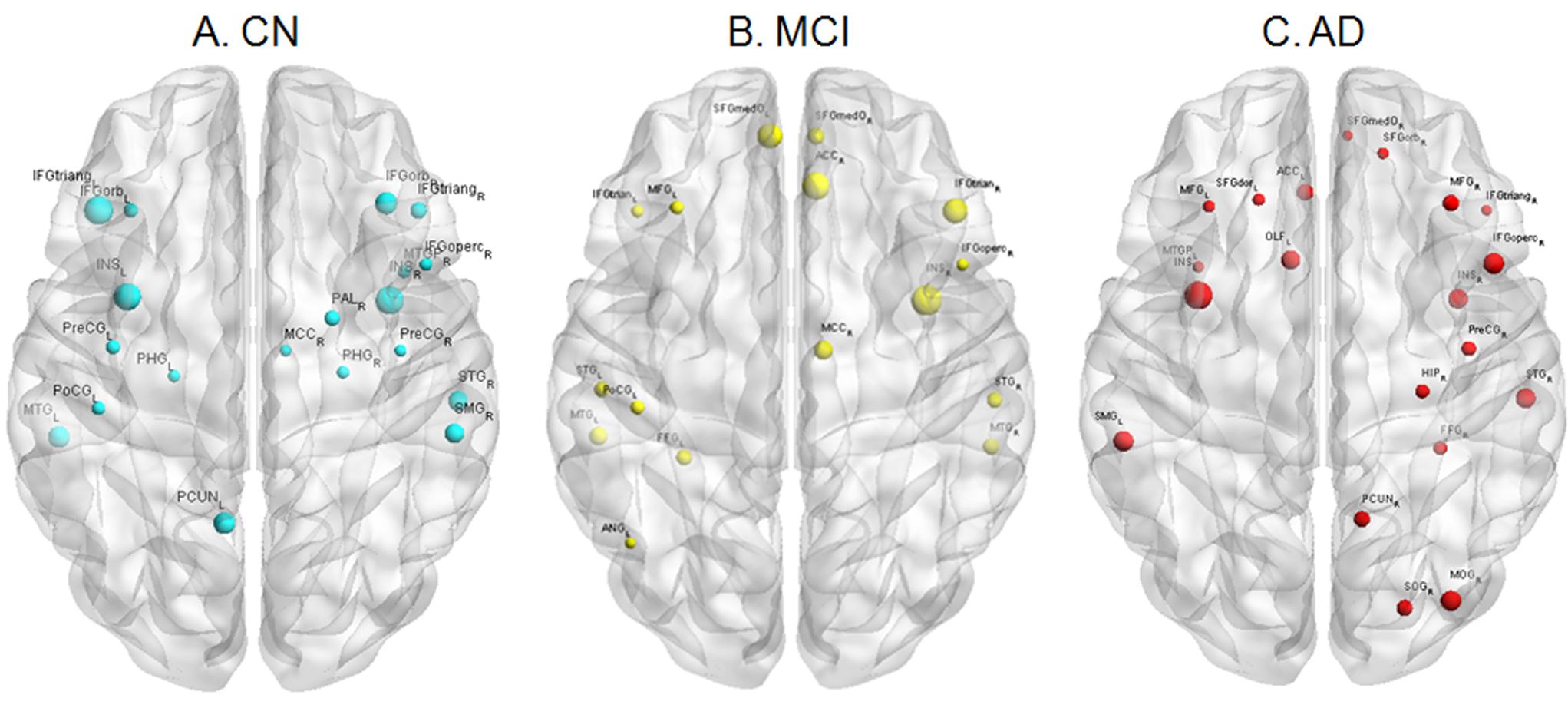

Supplement: Figure S1 — Functional hubs in CN, MCI, and AD. Normalized betweenness centrality >1.5 were considered as a hub. Node size corresponds with its value of normalized betweenness centrality. Hubs were visualized using the BrainNet viewer (NKLCNL, Beijing Normal University). CN = normal control; MCI = mild cognitive impairment; AD = Alzheimer’s disease. (TIF) [file pone.0053922.s001.tif]
