# Supplementary material for: Whole-brain Functional Networks in Cognitively Normal, Mild Cognitive Impairment, and Alzheimer’s Disease
Source: PLoS One. 2013 Jan 15;8(1):e53922. doi: 10.1371/journal.pone.0053922 (PMC3545923; doi:10.1371/journal.pone.0053922)
Supplement: Table S1 — Mathematical definitions of network parameters used in the study [20]. (DOC) [file pone.0053922.s002.doc]

| Table S1. Mathematical definitions of network parameters used in the study | | |
| --- | --- | --- |
| Parameter | Definitions | Mathematical expression |
| Degree, *k_i_* | The number of connections that link it to the rest of the network | | where 𝑁 is the set of all nodes in the network, 𝑎𝑖𝑗 is the connection status between 𝑖 and 𝑗. When an edge exists between these two nodes, 𝑎𝑖𝑗=1, otherwise 𝑎𝑖𝑗 =0   |  | | --- | |  | | | --- | --- | --- | |  | |
| Clustering Coefficients, *C_p_* | the average *C_i_* from entire nodes in the network, where *C_i_* is the likelihood that neighbors of a node will also be connected to each other | where *n* is the number of nodes, *t_i_* is number of triangles around node *i* and *C_i_* is Clustering Coefficients of node i (*C_i_* =0 for *k_i_* <2) |
| Characteristic path length, *L_p_* | the mean minimum number of edges of the shortest path connecting any two nodes |  |
|  |  | Where *d_ij_* is shortest path length (distance) between 𝑖 and 𝑗 and 𝐿𝑖 is the average distance between node 𝑖 and all other nodes |
| Small-worldness, σ  Betweenness centrality, *B_i_* | A network is considered as a small-world network if it show much higher *C_p_* while similar *L_p_* in comparison with the matched random networks  the number of shortest paths between any two nodes that run through node *_i_* | | 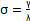 where γ=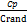, λ=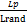,   | *C_p_* and 𝐶_rand_ are the clustering coefficients, and *L_p_* and 𝐿_rand_ are the characteristic path lengths of tested network and a random network respectively. Small-world networks often have  σ ≫1. | | --- | |  | | | --- | --- | --- | |  |   where *ρ_h_*_𝑗_ is the number of shortest paths between *h* and *j*, and 𝜌*_h_*_𝑗_(𝑖) is the number of shortest paths between *h* and *j* that pass through *i.* |
|  |  |  |
