# Supplementary material for: Whole-brain Functional Networks in Cognitively Normal, Mild Cognitive Impairment, and Alzheimer’s Disease
Source: PLoS One. 2013 Jan 15;8(1):e53922. doi: 10.1371/journal.pone.0053922 (PMC3545923; doi:10.1371/journal.pone.0053922)
Supplement: Table S2 — Functional hubs and nodal characteristics in normal control, mild cognitive impairment, and Alzheimer’s disease. (DOC) [file pone.0053922.s003.doc]

| Table S2. Functional hubs and nodal characteristics in normal control, mild cognitive impairment, and Alzheimer’s disease | | | |
| --- | --- | --- | --- |
| Region | *b_i_* | *k_i_* | *C_i_* |
| CN |  |  |  |
| IFGtriang_L | **4.01^*^**^†^ | 21 | 0.34 |
| INS_R | 3.96 | 21 | 0.32 |
| INS_L | **3.84^*^** | 19 | 0.35 |
| IFGorb_R | **3.10**^†^ | 24 | 0.38 |
| MTG_L | **3.07**^†^ | 13 | 0.32 |
| PCUN_L | **3.03^*^**^†^ | 21 | 0.60 |
| STG_R | 2.75 | 16 | 0.35 |
| SMG_R | **2.74^*^** | 18 | 0.41 |
| IFGtriang_R | 2.31 | 20 | 0.41 |
| PAL_R | 2.16 | 12 | 0.35 |
| IFGorb_L | 2.03 | 14 | 0.36 |
| PreCG_L | 2.02 | 25 | 0.50 |
| PoCG_L | 1.89 | 24 | 0.52 |
| MTGP_R | **1.82^*^** | 10 | 0.29 |
| PHG_R | **1.79^*^** | 15 | 0.39 |
| IFGoperc_R | 1.78 | 17 | 0.44 |
| PHG_L | **1.75^*^** | 14 | 0.42 |
| PreCG_R | 1.69 | 24 | 0.53 |
| MCC_R | 1.64 | 12 | 0.55 |
| MCI |  |  |  |
| INS_R | 4.28 | 18 | 0.31 |
| ACC_R | **3.85**^†‡^ | 7 | 0.43 |
| MFGorb_L | **3.51**^†‡^ | 16 | 0.42 |
| IFGtriang_R | **3.49^†^** | 19 | 0.43 |
| MCC_R | **2.71** | 4 | 0.33 |
| MTG_L | **2.59^†^** | 22 | 0.41 |
| MFGorb_R | **2.41**^‡^ | 16 | 0.47 |
| MTG_R | 2.25 | 16 | 0.38 |
| FFG_L | **2.23**^‡^ | 11 | 0.35 |
| STG_L | 2.17 | 21 | 0.41 |
| STG_R | 2.16 | 18 | 0.40 |
| PoCG_L | **1.99^†^** | 24 | 0.45 |
| MFG_L | 1.92 | 18 | 0.39 |
| IFGtriang_L | 1.77 | 17 | 0.49 |
| IFGoperc_R | 1.70 | 18 | 0.41 |
| ANG_L | **1.53^†^** | 19 | 0.37 |
| AD |  |  |  |
| INS_L | **3.98^*^** | 20 | 0.36 |
| IFGoperc_R | 2.95 | 24 | 0.39 |
| MOG_R | **2.90^‡^** | 22 | 0.40 |
| SMG_L | **2.86^*^** | 17 | 0.60 |
| STG_R | 2.82 | 17 | 0.38 |
| INS_R | 2.80 | 17 | 0.47 |
| OLF_L | **2.71^‡^** | 19 | 0.35 |
| ACC_L | 2.37 | 15 | 0.41 |
| MFG_R | 2.33 | 17 | 0.46 |
| SOG_R | 2.29 | 20 | 0.43 |
| PCUN_R | 2.26 | 14 | 0.44 |
| PreCG_R | 2.08 | 16 | 0.45 |
| HIP_R | **2.00^*‡^** | 9 | 0.67 |
| FFG_R | 1.96 | 10 | 0.33 |
| SFGdor_R | 1.77 | 17 | 0.34 |
| MFG_L | 1.74 | 18 | 0.48 |
| MTGP_L | **1.67^*‡^** | 7 | 0.29 |
| SFGorb_R | 1.65 | 19 | 0.52 |
| IFGtriang_R | 1.52 | 18 | 0.50 |
| MFGorb_R | 1.52 | 23 | 0.46 |

Normalized betweeness centrality >1.5 were listed as hubs in a descending order in each group.

^*^Significantly higher *b_i_* compared to MCI; ^†^Significantly higher *b_i_* compared to AD; ^‡^Significantly higher *b_i_* compared to CN

CN= cognitively normal; MCI= mild cognitive impairment; AD= Alzheimer’s disease; *b_i_* = normalized betweeness centrality of node i; *k_i_* = degree of node I; *C_i_* = regional clustering coefficient of node i Abbreviations for the regions are expanded in Table 1.
